# Long-Term Changes of Inflammatory Biomarkers in Individuals on Suppressive Three-Drug or Two-Drug Antiretroviral Regimens

**DOI:** 10.3389/fimmu.2022.848630

**Published:** 2022-03-14

**Authors:** Sergio Serrano-Villar, María Rosa López-Huertas, Daniel Jiménez, Carlos Galera, Javier Martínez-Sanz, Elena Moreno, Alfonso Muriel, Félix Gutiérrez, Carmen Busca, Joaquín Portilla, Otilia Bisbal, José Antonio Iribarren, Francisco Tejerina, Ignacio de los Santos, Santiago Moreno

**Affiliations:** ^1^Department of Infectious Diseases, Hospital Universitario Ramón y Cajal, Facultad de Medicina, Universidad de Alcalá, Instituto de Investivestigación Ramón y Cajal (IRYCIS), Madrid, Spain; ^2^CIBER de Enfermedades Infecciosas CIBER-Infec, Instituto de Salud Carlos III, Madrid, Spain; ^3^Centro Nacional de Microbiología, Instituto de Salud Carlos III, Madrid, Spain; ^4^HIV Unit, Hospital Universitario Virgen de la Arrixaca, Murcia, Spain; ^5^Biostatistics Unit, Hospital Universitario Ramón y Cajal, Facultad de Medicina, Universidad de Alcalá, IRYCIS, Madrid, Spain; ^6^CIBER Epidemiología y Salud Pública (CIBERESP), Instituto de Salud Carlos III, Madrid, Spain; ^7^Hospital General Universitario de Elche and Universidad Miguel Hernández, Alicante, Spain; ^8^HIV Unit, Hospital Universitario La Paz, Madrid, Spain; ^9^Department of Internal Medicine, Hospital General Universitario de Alicante, Alicante, Spain; ^10^HIV Unit, Hospital Universitario Doce de Octubre, Madrid, Spain; ^11^HIV Unit, Hospital Universitario de Donostia, Donostia, Spain; ^12^HIV Unit, Hospital Universitario Gregorio Marañón, Madrid, Spain; ^13^HIV Unit, Hospital Universitario La Princesa, Madrid, Spain

**Keywords:** antiretroviral therapy, inflammation, d-dimer, c reactive protein, HIV

## Abstract

**Background:**

Because inflammation is associated with mortality and has been linked to HIV transcription in lymphoid tissues during ART, it is necessary to address the long-term effects of switching 3-drug (3DR) to 2-drug regimens (2DR) on inflammation.

**Methods:**

Nested study in the Spanish AIDS Research Network. We selected PWH ART-naive initiating 3DR who achieved viral suppression in the first 48 weeks and either remained on 3DR or switched to 2DR (3TC+bPI; 3TC+DTG; DTG+RPV). We assessed the trajectories on inflammatory markers during ART using multivariate piecewise mixed models.

**Results:**

We analyzed 619 plasma samples from 148 patients (3DR, N=90; 2DR, N=58), the median follow-up was 4.6 (IQR 3.2-6.2) years. There were no significant differences in baseline characteristics between groups. After adjusting for potential confounders, patients with 3DR experienced a slow decline of IL6, hs-CRP, sCD14, sCD163, and D-dimer over time. In contrast, compared to 3DR, switching to 2DR was associated with increases in IL-6 (p=0.001), hs-CRP (p=0.003), and D-dimer (p=0.001) after year 3 from virologic suppression. 2DR was associated with a higher risk of hs-CRP quartile increase (aOR 3.3, 95%CI 1.1-10) and D-dimer quartile increase (aOR 3.7, 95%CI 1.1-13). The adjusted biomarker trajectories did not reveal a distinct pattern according to the type of 2DR used (bPI *vs* DTG).

**Conclusions:**

In this study in virally suppressed individuals, maintaining 3DR was associated with a more favorable long-term inflammatory profile than switching to 2DR. The potential clinical implications of these findings on the development of non-AIDS events deserve further investigation.

## Introduction

Levels of inflammatory biomarkers remain increased in people with HIV (PWH), even when antiretroviral therapy (ART) is started early (1, 2). This has consistently been associated with an excess risk of comorbidities during HIV treatment and suggested as a contributing risk factor (3–5). Inflammation is also linked to HIV transcription and translation into viral proteins within lymphoid tissues during antiretroviral therapy (ART)-mediated viral suppression (6–8). Hence, it is necessary to address the long-term effects of reducing the number of drugs in current HIV therapeutic schemes on inflammation.

While the virologic efficacy and safety of current ART regimens are difficult to improve, a major challenge is to fully normalize the health of PWH. Non-virologic parameters associated with excess risk of mortality, such as increased bacterial translocation or inflammation (9, 10), are often assumed to be a consequence of the immune damage elicited by acute HIV infection, but no longer affected by HIV replication in individuals on ART. However, even during HIV RNA suppression in PWH, a lower ART adherence to 3-drug regimens (3DR) is associated with increased levels of inflammatory biomarkers, suggesting that a decrease in drug concentrations results in virologic phenomena in tissues, not detected in plasma, that elicit immune activation (11–13). Mounting evidence generated in SIV-infected macaques, HIV-infected humanized mice, and humans indicates that antiretrovirals are distributed very heterogeneously within lymphoid tissues (14, 15). Remarkably, the largest amount of intracellular HIV RNA is expressed in areas with no drug exposure, arguing PK/PD phenomena explain in part the persistence of low-level replication within tissues (15).

Because inflammation is slow to change in virally suppressed individuals (16), studies aiming to assess inflammatory biomarkers are challenged by the short period evaluated. Clinical trials powered to detect differences between ART strategies on the development of non-AIDS comorbidities will likely not be performed in the future. Cohort studies can provide valuable information on the long-term dynamics of inflammatory markers during different ART strategies.

Here, we performed a nested study in the Spanish AIDS Research Network (CoRIS). We asked whether switching to 2DR is associated to a different long-term inflammatory profile compared to remaining on 3DR.

## Methods

### Study Design, Participants, Setting, and Eligibility

We performed a nested study in the Cohort of the Spanish HIV Research Network (CoRIS)—a national ongoing prospective multicenter cohort of people with HIV (PWH), including 14,458 treatment-naïve adults recruited from 45 Spanish hospitals. We included ART naïve patients who initiated follow-up between January 2^nd^, 2004, and December 28^th^, 2018, who were included in a prospective clinical cohort study with continuous enrollment and standardized data collection (17). CoRIS is a joint activity of the Research Network of Excellence that incorporates basic scientists, virologists, immunologists, clinicians, epidemiologists, and statisticians. This database collects demographic and clinical data, HIV transmission category, ART history, prior opportunistic diseases, comorbidities, serologic and immunovirological data, and specific data on non-AIDS events. Internal quality controls are performed annually, and 10% of data are externally audited every two years. Samples from patients were kindly provided by the HIV BioBank integrated in the Spanish AIDS Research Network (18). Samples were processed following current procedures, aliquoted, and frozen immediately after their reception. All patients participating in the study gave their informed consent and protocols were approved by institutional ethical committees.

We selected ART naïve patients initiating 3DR from 2004 to 2008 with either boosted protease inhibitor (bPI)- or integrase strand transfer inhibitor (INSTI)-based combinations, who achieved virologic suppression in the first 48 ± 6 weeks of ART and who either remained on their selected 3DR or switched to 2DR consisting of lamivudine plus either a bPI or dolutegravir or of rilpivirine and dolutegravir, or to monotherapy (MT) with either lopinavir/ritonavir (LPV/r) or boosted darunavir (bDRV) after at least 48 weeks or virologic suppression. The decision to exclude non-nucleoside reverse transcriptase inhibitor (NNRTI)-based first-line ART was made to avoid the potential confounding effects of the third-drug used in 3DR in the comparison against 2DR or MT. Participants maintained virologic suppression until the last plasma sample available at the biobank and had available stored samples at the following timepoints: before ART initiation, after achieving virologic suppression, and at least two additional plasma samples with the treatment evaluated (3DR, 2DR or MT). Exclusion criteria were the presence of virologic failure during the first 48 weeks of ART and the diagnosis of AIDS and serious non-AIDS events (malignancies, cardiovascular diseases, end-stage liver disease, and end-stage kidney disease) before ART initiation of during the first 48 weeks of ART. We defined virologic failure as two consecutive HIV RNA measurements >50 copies/mL, HIV RNA blips as a single HIV RNA measurement between 50 and 1000 copies/mL, and virologic failure with low-level viremia as two consecutive measurements between 50 and 1000 copies/mL.

The Institutional Review Boards of the Carlos III Health Institute located in Madrid, Spain, the Ethics Committee at University Hospital Ramón y Cajal approved the study (ceic.hrc@salud.madrid.org, approval number 133-17).

### Sample Size

Of the 14,458 individuals included in CoRIS, 7,655 had started ART with 3DR, 4,613 had at least one sample available in the first 96 weeks after achieving the virologic suppression, and 1,000 met the sample availability requirements. After virologic suppression, 705 individuals maintained their 3DR regimen and maintained the virologic suppression until the last available plasma sample. From the 4,613 individuals, 255 and 199 switched to 2DR and MT during follow-up, 196 and 88 maintained the selected regimen over time, 62 and 31 had at least one plasma sample stored under the same 2DR or MT treatment regimen, and 58 and 23 had enough plasma volume stored for the biomarker measurements. To meet the criteria of availability of at least two plasma samples on the evaluated regimen, we requested the centers participating in CoRIS to send an additional plasma sample to the biobank in those cases with only a single observation under the evaluated regimen or with a last sample sent long before the end of the follow-up.

### Measurement of Plasma Cytokines

We measured levels of D-dimer, IL6, hs-CRP, IFABP, sCD14, and sCD163 in plasma as indicators of coagulation, inflammation, monocyte activation, and bacterial translocation using the commercial ELISA assays Human D2D (D-Dimer) ELISA Kit (ImmunoWay Biotechnology, Plano, Texas), Human Quantikine IL-6 HS ELISA (R&D Systems, Minneapolis, Minnesota), CRP ELISA (LDN Labor Diagnostika Nord, Nordhorn Germany), PicoKine ELISA for Human IFABP (Boster Bio, Pleasanton, California), Human CD14 Quantikine ELISA Kit (R&D Systems) and Human CD163 Quantikine ELISA Kit (R&D Systems), respectively, accordingly to manufacturers’ instructions. Each sample was assayed in duplicate in batches seeking an equal representation of the study groups and containing all samples from the same individual. Data was collected using a microplate luminometer with Gen5 Data Analysis Software (Biotek, Winooski, Vermont).

### Sample Size and Power Calculation

We used IL-6 as the primary outcome variable for sample size calculation. We considered the IL-6 distribution data from a previous study in PWH. (19) According to the longitudinal study by Wada et al. (16), the yearly percentage change of IL-6 during the first year after HIV suppression is -13%, and -1% during each year following the first year of HIV suppression. Hence, assuming a median follow-up of 3 years, we expected for 3DR at least a 15% decrease from the baseline to the last timepoint. We assumed that a 20% difference in the median change of inflammatory biomarkers from baseline to censoring would be clinically relevant. Thus, for a power of 80% and an alpha error of 0.05, a delta of 20%, and a sample size in the experimental group (2DR) that is half of the control group (3DR), we would need 32 individuals in each group. Since there were only 23 individuals in the MT group, the 3DR *vs.* MT comparisons are exploratory. Similarly, due to the smaller sample sizes we did not compute the statistical significance of the *posthoc* analyses segregating by the type of 2DR.

### Statistical Analysis

According to the variable distribution, qualitative variables were reported as frequency distribution, whereas we described quantitative variables using medians with their interquartile ranges (IQR) or means with their standard deviations (SD). We fixed linear mixed models for the primary analysis to allow for correlation due to repeated measurements on the same subject. Interaction terms (time-versus-treatment group) were created to assess whether these changes differed significantly over time between the treatment groups. When the observed trajectories were not linear, we used piecewise linear mixed models to compare the slope parameters between treatment groups at the intervals defined by the inflection points. Because the median time to ART switch was three years from virologic suppression and there was an inflection point at this timepoint, we compared the slope parameters between treatment groups at year 0-3, and 3-8 from virologic suppression. To illustrate the predicted adjusted changes of inflammatory biomarkers, we plotted the values predicted by the piecewise models at the intervals between years 0, 1, 2, 3, 4, and 8, as previously performed in CoRIS for comparison of treatment effects on continuous variables (20). Analyses were adjusted for age, sex, mode of transmission, education level, calendar year, baseline HIV RNA, diagnosis of AIDS during follow-up, nadir CD4, and inflammatory biomarker concentration at HIV RNA suppression.

We assessed the probabilities of biomarker quartile increase over time in each group using multivariate logistic regression models, adjusted for the same covariates described above. We explored the crossed correlations between biomarkers by calculating Spearman’s Rho coefficients. Continuous outcome variables were log-transformed when necessary to satisfy model assumptions. We used Stata v. 17.0 (StataCorp LP College Station, TX, USA) for all statistical analyses.

## Results

Of the 14,458 individuals included in CoRIS, 11,330 started ART with a combination of two nucleoside reverse-transcriptase inhibitor (NRTI) and either one NNRTI, one PI, or one INSTI; and 9,282 had achieved undetectable plasma HIV RNA in the first 48 ± 6 weeks of ART initiation.

During the follow-up, 7,665 individuals remained on 3DR, 424 changed to 2DR, and 327 changed to MT. The general characteristics of the whole cohort are provided in **Table S1**. A total of 148 individuals had at least 3 stored plasma samples after HIV RNA suppression and were selected, including 90 patients with 3DR, 58 with 2DR, and 23 with MT. Because of the smaller sample size of the MT group, we report the analyses in the **Supplemental Material**.

The median follow-up was 4.3 years (IQR 3.0-6.2), and the median number of samples analyzed was 4 (IQR 3-11), accounting for 680 observations. The study sample was representative of a medium-aged population of individuals with HIV with a higher representation of men. **Table 1** shows the population characteristics. Patients in each group had a similar socio-demographic profile, without statistically significant differences in their baseline characteristics, except for a higher maximum HIV RNA in the 3DR group compared to the MT group (p=0.077) and a lower nadir CD4 count in the 3DR group compared to the MT group (p=0.061). The median time from virological suppression to ART change in the 2DR and MT groups was 3 years (IQR 1.6 – 4.9).

**Table 1:** Population baseline characteristics according to ART regimen.

	Patients remaining on 3DR	Patients changing to 2DR	p value
	N=90	N=58	
**Age (mean**, [**SD])**	37 (9)	40 (11)	0.106
**Gender, n (%)**			0.936
Male	78 (87)	50 (86)	
Female	12 (13)	9 (14)	
**Mode of transmission, n(%)**			0.972
MSM	69 (67)	38 (65)	
Heterosexual	21 (23)	15 (26)	
IDU	6 (7)	15 (26)	
Unknown	3 (3)	2 (3)	
**Spanish Origin, n (%)**	59 (66)	36 (62)	0.666
**Education level, n (%)**			0.748
No studies or compulsory	13 (14.4)	4 (17.4)	
Upper secondary or university	64 (71.1)	17 (73.9)	
Unknown	13 (14.4)	2 (8.7)	
**AIDS diagnosis, n (%)**	14 (16)	8 (14)	0.769
**HCV positive ever, n (%)**	12 (13)	6 (10)	0.615
**Maximum HIV-1 RNA (c/mL), median (IQR)**	114500 (33770-344426)	93599 (36307-219000)	0.377
**< 100000, n (%)**	42 (46.7)	32 (55.2)	0.312
**≥ 100000, n (%)**	48 (53.3)	26 (44.8)	
**Time from ART initiation to virologic suppression (years), median (IQR)**	0.5 (0.2-0.9)	0.5 (0.3-0.9)	0.126
**Virologic failure ever, n (%)**	6 (6.7%)	1 (1.7%)	0.167
**Low level viremia ever, n (%)**	4 (4.4%)	0 (0%)	0.104
**Time from virologic suppression (years) to ART switch, median (IQR)**	–	3.5 (1.9-5.2)	–
**Nadir CD4 cell count (cells/μL), median (IQR)**	300 (151-373)	259 (112-382)	0.309
**Number of samples analyzed, median (min, max)**	4 (3-11)	3 (3-8)	< 0.001
**Follow-up (years), median (IQR)**	3.9 (2.5-4.7)	5.3 (3.9-6.8)	< 0.001

TT, triple therapy; 2DR, 2-drug combinations; ART, antiretroviral therapy; IDU, injecting drug use; MSM, men who have sex with men.

**Figure 1:**
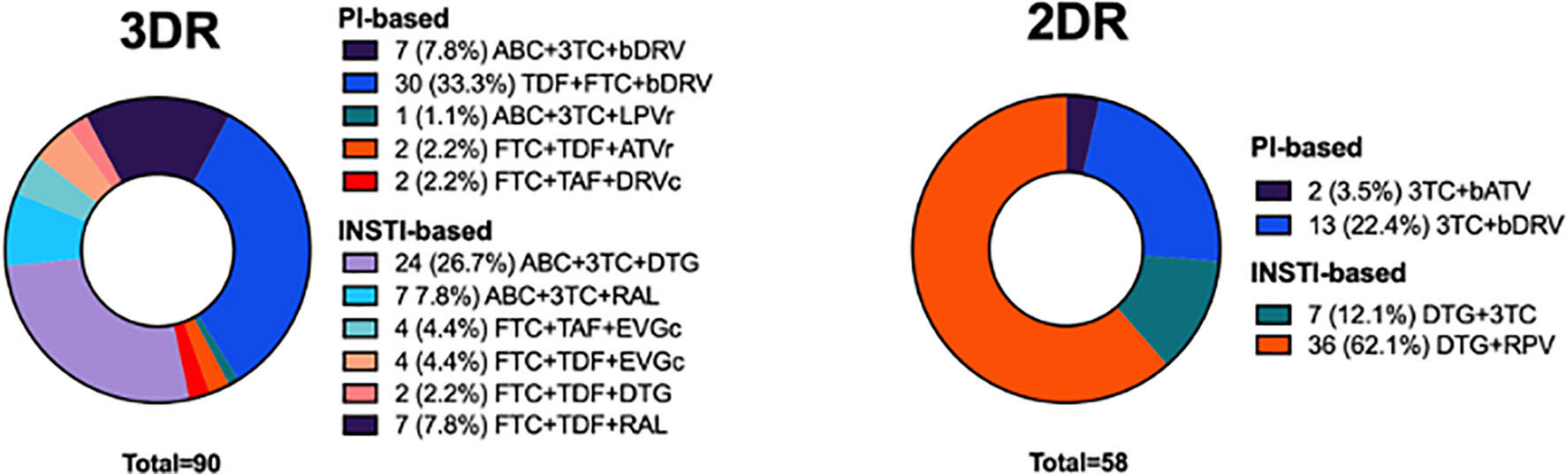
Details the distribution of ART combinations in the study population.

During follow-up, there were 5 (5.6%) instances of virologic failure after the last available plasma sample with 3DR, 1 (1.6%) in 2DR, and 0 in MT (p=0.270), and 4 (1.1%) instances of low-level viremia with 3DR, 0 in 2DR and 0 in MT (p=0.166). The occurrence of severe non-AIDS events during follow-up was rare in the cohort, and no differences between groups were apparent (**Table S2**). The proportion of patients receiving INSTI-based ART during the evaluation period was lower in the 3DR group (48 patients, 53%) than in the 2DR group (43 patients, 75%)(p=0.011), of whom the majority were receiving dolutegravir and lamivudine (**Figure 1**).

### Effects of 2-Drug Regimens (2DR) on Linear Trajectories of Inflammatory Biomarkers

**Figure 2:**
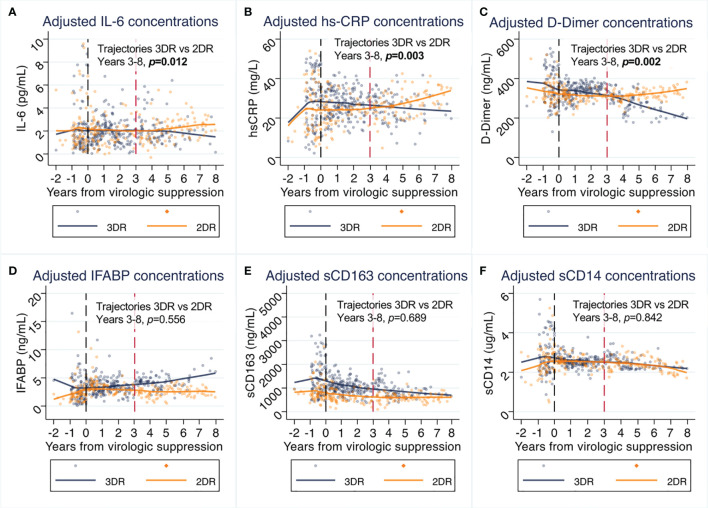
**(A–F)** Effects of switch to 2-drug regimens (2DR) on inflammatory biomarkers compared to remaining on 3-drug regimens (3DR). Panels represent the values predicted in piecewise linear mixed models at intervals between years 0-1, 1-2, 2-3, 3-4 and 4-8, adjusted for age, sex, country of origin, mode of transmission, educational level, maximum HIV RNA, previous AIDS, nadir CD4, and pre-ART biomarker value. Lines represent predicted mean values and dots the individual observations. The P values shown in represent the between-group comparison of biomarker trajectories from year 3 to 8. The tables piecewise comparisons for the periods 0-3 and 3-8, are provided in **Table 2**. 3DR, 3-drug regimens; 2DR, 2-drug regimens.

**Figure 2** shows the linear trajectories of the biomarkers assessed in the 3DR *vs.* 2DR groups after adjusting for all covariates in the linear mixed models (**Table 2**).

**Table 2:** Adjusted coefficients of inflammatory biomarker changes by ART strategy (2-drug regimens (2DR) *vs.* 3-drug regimens (3DR)) at year 0-3 and 3-8 from virologic suppression.

	Coefficient	95% CI	p
**Years 0-3 of viral suppression**			
IL-6 (log10)	-0.025	-0.123, 0.072	0.611
hs-CRP (log10)	-0.002	-0.111, 0.107	0.969
D-dimers	-18.474	-39.430, 2.483	0.084
IFABP	-0.333	-0.105, 0.385	0.364
sCD163 (log10)	-0.007	-0.065, 0.049	0.787
sCD14	-0.056	-0.279, 0.167	0.623
**Years 3-8 of viral suppression**			
IL-6 (log10)	0.103	0.022, 0.182	**0.012**
hs-CRP (log 10)	0.0003	0.0001, 0.005	**0.003**
D-dimers	30.888	11.317, 50.460	**0.002**
IFABP	-0.199	-0.860, 0.462	0.556
sCD163 (log10)	0.011	-0.044, 0.067	0.690
sCD14	0.0165	-0.146, 0.179	0.843

*Reference category: 2DR

Each coefficient represents a separate analysis adjusted for were adjusted for age, sex, mode of transmission, education level, calendar year, baseline HIV RNA, diagnosis of AIDS during follow-up, nadir CD4, and inflammatory biomarker concentration at HIV RNA suppression.

ART, antiretroviral therapy; 3DR, triple therapy; 2DR, 2-drug combinations; CI, confidence interval; INSTI, integrase strand transfer inhibitor; NRTI, nucleoside reverse transcriptase inhibitor; NNRTI, nonnucleoside reverse transcriptase inhibitor; PI, protease inhibitor.

Bold denote the values with p<0.05.

**Figure 3:**
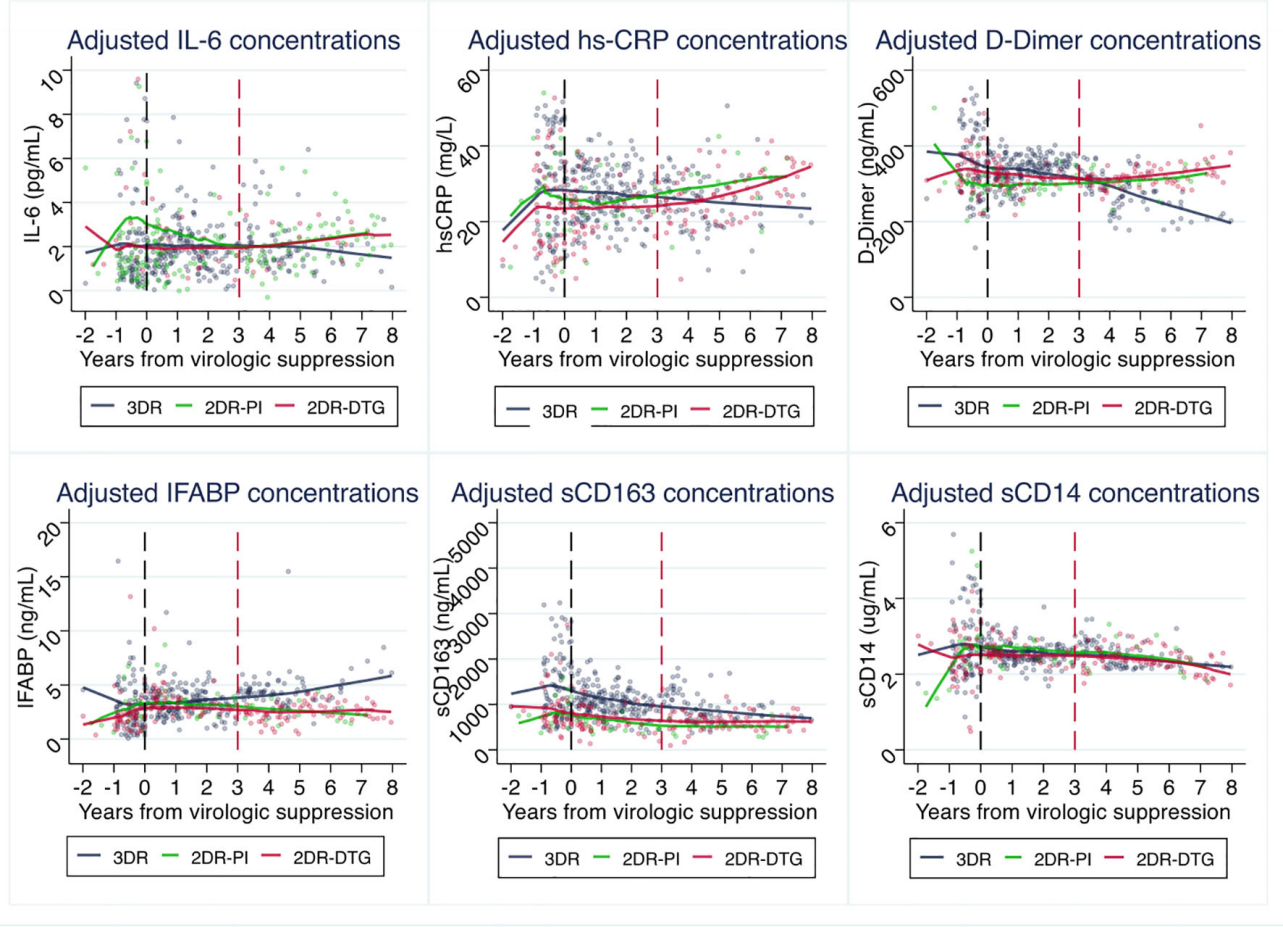
Effects of switch to 2-drug regimens (2DR), segregated by the type of 2DR regimen, on inflammatory biomarkers compared to remaining on 3-drug regimens (3DR). Panels represent the values predicted in piecewise linear mixed models at intervals between years 0-1, 1-2, 2-3, 3-4 and 4-8, adjusted for age, sex, country of origin, mode of transmission, educational level, maximum HIV RNA, previous AIDS, nadir CD4, and pre-ART biomarker value. Lines represent predicted mean values and dots the individual observations. Abbreviatures, 3DR, 3-drug regimens; 2DR-PI, protease inhibitor 2-drug regimens; 2DR-DTG, dolutegravir-based 2-drug regimens.

We did not appreciate differences in the pattern of changes of IFABP, sCD163, and sCD14 changes over time. However, the adjusted trajectories of IL-6, hs-CRP, and D-dimer diverged during follow-up between groups after year three from virologic suppression. While in the 3DR group IL-6, hs-CRP and D-dimer decreased over time, there was an inflection point at year 3 from virologic suppression, leading to increases in these biomarkers with 2DR *vs.* 3DR (IL-6 p=0.012, hs-CRP p=0.003, and D-dimer p=0.002 for the trajectories after year 3). Subanalyses segregating the curves by the core agent used as 2DR (bPI or INSTI) indicated that the effect was not dependent on the type of 2DR used (**Figure 3**).

Overall, the trajectories of the inflammatory biomarkers in the MT group followed a pattern comparable to the 2DR group (**Figure S2**). However, the smaller sample size in this group limited the statistical power to detect differences, and no statistically significant differences were found from year 3 to 8 (**Table S3**).

### Effects of Switching to 2DR on Biomarker Quartile Increase

**Figure 4:**
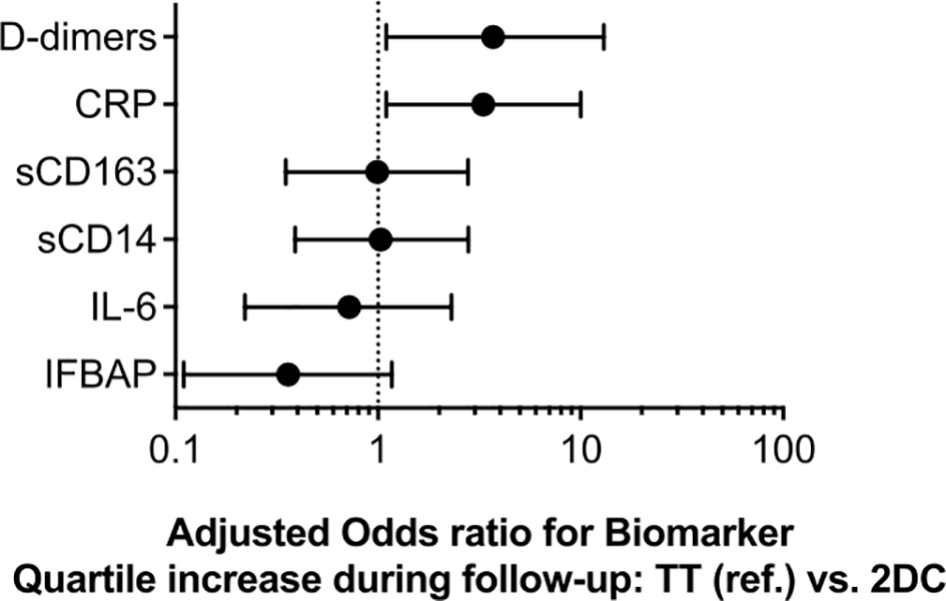
Adjusted odds ratio for biomarker quartile increase during follow-up by associated with switch to 2-drug regimens (2DR) compared to remaining on 3-drug regimens (3DR). Models were adjusted for age, sex, mode of transmission, education level, calendar year, baseline HIV RNA, diagnosis of AIDS during follow-up, nadir CD4, inflammatory biomarker concentration at HIV RNA suppression.

Because previous works investigating the links between inflammatory markers and clinical progression have investigated the association as excess mortality per quartile change (21, 22), we next explored the effect of treatment on the risk of quartile increase over time. We found that the switch to 2DR was associated with a higher probability of D-dimer (aOR 3.3, CI95% 1.1 – 10, p=0.032) and hs-CRP (aOR 3.7, CI95% 1.1 – 12, p=0.029) quartile increase during follow-up. No differences were detected for the other biomarkers (**Figure 4**).

### Crossed-Correlations Between Inflammatory Biomarkers

**Figure 5:**
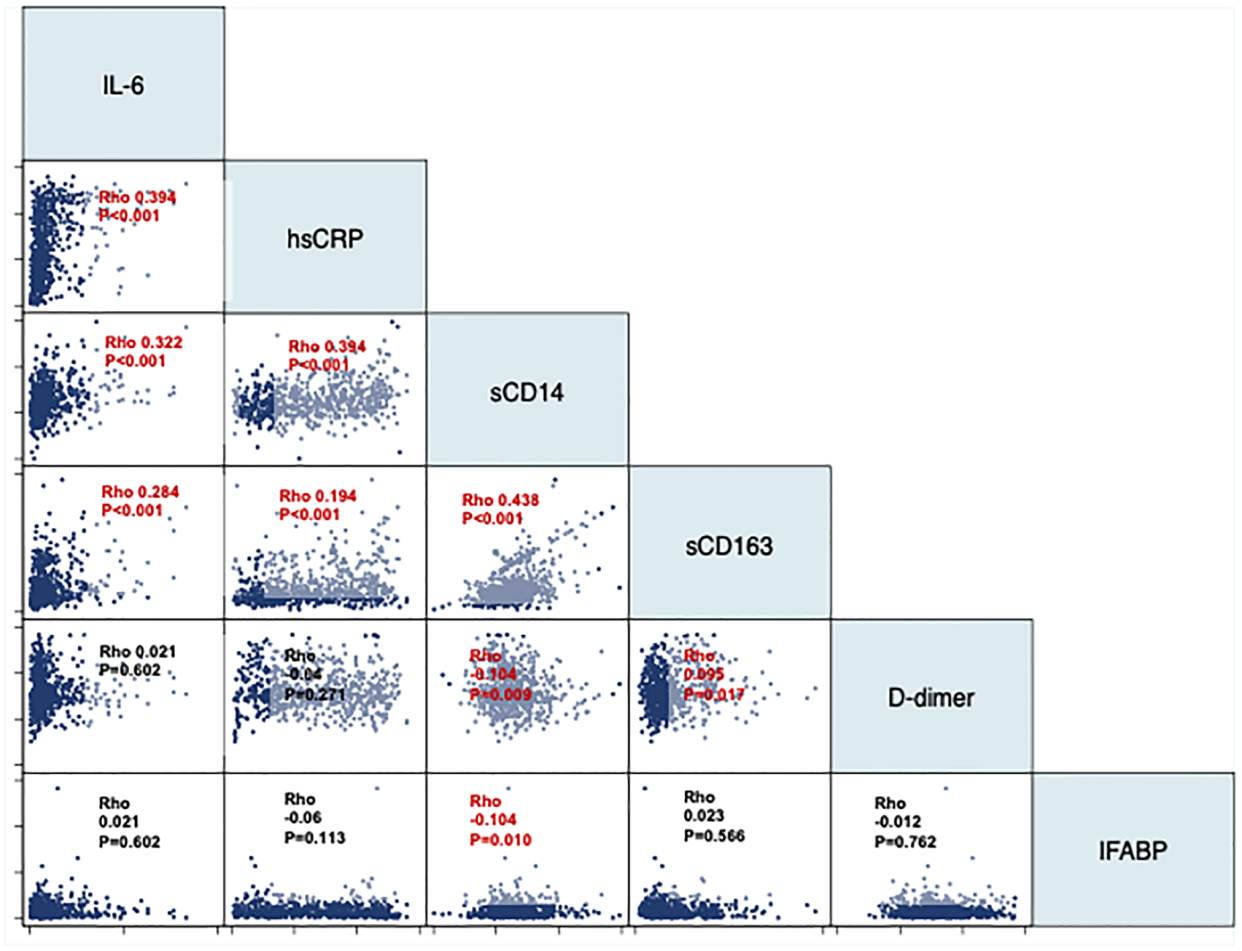
Pairwise correlations between inflammatory biomarkers. The panel represent pairwise correlations using Spearman’s Rho coefficients between each pair of inflammatory biomarkers.

To gain information on the relatedness of the assessed biomarkers, we calculated the Spearman’s Rho pairwise correlations between them. In general, the assessed biomarkers showed weak (Rho <0.3) but statistically significant correlations (**Figure 5**), arguing that each one reflects independent biological pathways. sCD163 and sCD14 were the more strongly correlated (Rho 0.438, p<0.001).

## Discussion

This was a nested study within a large prospective cohort of PWH on 3DR either maintaining 3DR or switching to 2DR or MT. We found that maintaining 3DR was associated with a more favorable long-term inflammatory profile than reducing the number of antiretrovirals in the regimen. These differences occurred regardless of adjustment for the main determinants of ART choice and the maintenance of HIV RNA suppression and affected three of the six assessed biomarkers, including IL-6, hs-CRP and D-dimer.

Inflammation is an extremely complex phenomenon. The lack of correlation between biomarkers indicates that each one represents an independent biological pathway, even for those belonging to the same inflammatory pathway, such as IL-6 and hs-CRP. A major challenge for the field is to further understand what the different inflammation patterns imply in terms of risk prediction. For example, as recently found in a large cohort study in the CFAR Network of Integrated Clinical Systems (CNICS) cohort, type 1 myocardial infarction, type 2 myocardial infarction, and deep vein thrombosis were predicted by distinct inflammatory patterns (23).

Despite the consistent link between inflammation and an excess risk of severe non-AIDS events and mortality (24, 25), the information regarding the long-term effects of current ART strategies on inflammation has been barely explored, probably because of the assumption that different ART strategies should not differently affect inflammation, provided that they efficiently suppress the virus in plasma. In addition, because inflammatory biomarkers change slowly in ART-suppressed individuals (16), the comparison of ART strategies on inflammation may need long follow-up to detect slight differences between treatment groups. Conceptually, the most likely mechanism by which a suppressive 2DR could yield an increase of plasma inflammatory biomarkers is related to the magnitude of HIV RNA expression and translation in locations where drugs are poorly distributed, mainly lymphoid tissue, which would then trigger the inflammatory response (11–13, 15). However, no studies have directly assessed this hypothesis.

We selected the inflammatory markers more robustly associated with the risk of severe non-AIDS events and mortality in HIV (23). Of note, higher levels of inflammatory (IL-6, hs-CRP) and coagulation (D-dimer) markers are associated with an increased risk of cardiovascular disease (CVD) (26), cancer (27), and all-cause mortality (28). IL-6 and D-dimer have been those more thoroughly evaluated in the HIV field, with consistent effect sizes even between studies on participants with high and low CD4 nadir (29). In the SWORD studies, participants who switched to dolutegravir and rilpivirine from different 3DR regimens showed 1.5-2 fold-increases of IL6, sCD163, and sCD14, and smaller decreases of VCAM and IFABP after 100 weeks of follow-up, with no changes on hs-CRP or D-dimer, compared to those who had switched to the same regimen 48 weeks later (30). After 148 weeks of follow-up, significant D-dimer increases, and IFABP reductions were observed in both groups (31). Because at these timepoints all participants were on 2DR, these data cannot clarify whether the number of drugs influences these biomarkers. A statistically significant 16% increase in IL-6 and a 3% reduction in sCD14 were observed in the TANGO study after 48 weeks of switch from tenofovir alafenamide-based 3DR to dolutegravir and lamivudine, compared to remaining in 3DR (32), that were also appreciated after 144 weeks (33). While the information generated in these clinical trials is overall reassuring, the increases in IL6 levels with 2DR observed TANGO, SWORD, and also in our observational study support the hypothesis that 3DR might exert stronger anti-inflammatory effects, and encourages additional investigation in this field.

The major strengths of our study include the prospective collection of data and samples and the long follow-up, with a median of 5 years in the 2DR group, and the number of samples analyzed per patient, which allowed controlling for intra-individual variation. Both factors increased the power of our study to detect differences between groups. In addition, we only included 2DR with efficacy is supported by randomized controlled trials. Finally, because the rationale to expect an effect largely relies on tissue drug distribution, if any, an effect would be more apparent in an observational study than in a controlled trial, where ART adherence is expected to be higher.

Our study also has several limitations that prompt a cautious interpretation of our results. First, there is still a risk of unmeasured confounding as with any observational study. Given the large sample size and follow-up and the prospective nature of CoRIS, we could adjust the analyses for the major determinants of ART choice, but could not for other potentially relevant confounders such as the presence of comorbidities or treatments that could affect inflammation. However, no significant differences were found in the general characteristics of each cohort at baseline. The fact that many research groups participating in CoRIS have focused their research on 2DR could explain that the general characteristics of individuals in the 2DR group did not significantly differ from those in the 3DR group. In addition, our findings were independent of the immunovirological covariates analyzed (i.e., CD4 nadir, maximum HIV RNA). The higher number of episodes of virologic failure and low-level viremia occurring after the last available sample analyzed suggests that adherence was lower in individuals with 3DR, as expected in an observational study in which clinicians would be less likely to reduce the number of drugs in less adherent patients. In addition, the limited sample size determines that the detected effects could have been dependent of a small number of observations. Another source of potential bias is the immortal time bias, which is inherent to observational studies comparing treatment interventions. Because a subject had to survive to switch to 2DR, but not to remain on 3DR, if this bias existed, it would have underestimated the real difference. The number of virologic failures during follow-up was higher with 3DR, which also suggests that patients remaining on 3DR were on average less adherent than those switching to 2DR. Also, we lacked information that would have helped to interpret the results, such as ART adherence or weight gain, that are expected to affect inflammation. Last, because of the insufficient information obtained in the MT group, we lacked of statistical power to provide more accurate effect estimates. As a planned analysis, we decide to report the 3DR *vs.* MT comparison, but be interpreted as an exploratory sub-analysis. However, the patterns observed in the MT group were consistent with those observed with 2DR.

While our study fuels the debate on the potential differences of ART strategies on inflammation, our data should be interpreted only as exploratory of the long-term effects on inflammatory biomarkers of certain ART combinations. Even if our results are reproduced in other cohorts and controlled studies, it would still be important to analyze the impact of these differences on the risk of clinical events, for which very large studies would be needed. However, because several inflammatory markers are easy to measure and independently predict the risk of severe non-AIDS events and mortality (3–5, 26–28), their measurement as exploratory endpoints in clinical trials will help advance our current understanding of the effect of ART strategies on inflammation. Evaluation of the impact of specific therapeutic interventions on long-term inflammation in prospective cohorts and clinical trials with mechanistic substudies is to further understand the differential long-term effects of ART combinations beyond virological suppression in plasma.

## Data Availability Statement

Due to ethical restrictions, some restrictions may apply. Study data requests should be addressed to the Spanish AIDS Cohort Research Network. Requests to access the datasets should be reasoned and directed to the corresponding authors.

## Ethics Statement

The studies involving human participants were reviewed and approved by Comité de investigación clínica del Hospital Universitario Ramón y Cajal (email: ceic.hrc@salud.madrid.org). Approval number 133-17. The patients/participants provided their written informed consent to participate in this study.

## Author Contributions

SS-V and SM conceptualized the study. SS-V analyzed the data. AM supervised the statistical analysis. SS-V generated the figures, and wrote the first draft of the manuscript. ML-H, DJ, and EM performed the laboratory measurements. SS-V, CG, JM-S, FG, FT, CB, OB, IS, JP, JI, and SM contributed to data mining. All authors revised and approved the final manuscript.

## Funding

The HIV BioBank, integrated in the Spanish AIDS Research Network, is supported by Instituto de Salud Carlos III, Spanish Health Ministry (Grant n° proyectos RD06/0006/0035, RD12/0017/0037 and RD16/0025/0019) as part of the Plan Nacional R + D + I and co-financed by the European Development Regional Fund ‘‘A way to achieve Europe’’ (ERDF). The RIS Cohort (CoRIS) is funded by the Instituto de Salud Carlos III through the Red Temática de Investigación Cooperativa en SIDA (RIS C03/173, RD12/0017/0018 and RD16/0002/0006) as part of the Plan Nacional R+D+I and cofinanced by ISCIII-Subdirección General de Evaluacion and Fondo Europeo de Desarrollo Regional (FEDER)”. This work was supported by the Instituto de Salud Carlos III projects AC17/00019, PI18/00154, ICI20/00058, CIBER de Enfermedades Infecciosas, and Gilead Sciences (Investigator Sponsored Research ISR-17-10192). The funders had no role in the study design, data analysis, or in the interpretation of the results

## Conflict of Interest

Outside the submitted work, SS-V reports personal fees from ViiV Healthcare, Janssen Cilag, Gilead Sciences, and MSD as well as non-financial support from ViiV Healthcare and Gilead Sciences and research grants from MSD and Gilead Sciences. JM-S, non-financial support from ViiV Healthcare, non-financial support from Jannsen Cilag, non-financial support from Gilead Sciences, outside the submitted work. JP reports grants from Instituto de Salud Carlos III during the conduct of the study; grants and personal fees from Gilead Sciences, personal fees from Janssen Cilag, personal fees from ViiV Health Care, personal fees from MSD, outside the submitted work. SM reports grants, personal fees and non-financial support from ViiV Healthcare, personal fees and non-financial support from Janssen, grants, personal fees and non-financial support from MSD, grants, personal fees and non-financial support from Gilead, outside the submitted work.

The remaining authors declare that the research was conducted in the absence of any commercial or financial relationships that could be construed as a potential conflict of interest.

The reviewer PH declared a past collaboration with one of the authors SS-V to the handling editor.

## Publisher’s Note

All claims expressed in this article are solely those of the authors and do not necessarily represent those of their affiliated organizations, or those of the publisher, the editors and the reviewers. Any product that may be evaluated in this article, or claim that may be made by its manufacturer, is not guaranteed or endorsed by the publisher.
