# Supplementary material for: Long-Term Changes of Inflammatory Biomarkers in Individuals on Suppressive Three-Drug or Two-Drug Antiretroviral Regimens
Source: Front Immunol. 2022 Mar 14;13:848630. doi: 10.3389/fimmu.2022.848630 (PMC8964183; doi:10.3389/fimmu.2022.848630)
Supplement: Supplementary file 1 [file DataSheet_1.pdf]

Long-Term Changes of Inflammatory Biomarkers in Individuals on Suppressive Three-drug or Two-drug Antiretroviral Regimens

Supplemental Materials

Supplemental tables

Table S1. Population baseline characteristics according to the ART regimen in the whole cohort.

	Subjects remaining on 3DR	Subjects changing to 2DR	Subjects changing to MT	p value
	**N=7665**	**N=424**	**N=327**	
**Age (mean, [SD])**	37 (30 – 44)	38 (31 – 45)	37 (30 – 45)	0.02
**Gender, n (%)**				0.009
Male	6480 (84.5)	323 (80.9)	260 (79.5)	
Female	1185 (15.5)	76 (19.1)	67 (20.5)	
**Mode of transmission, n(%)**				0.003
MSM	4698 (61.3)	236 (59.1)	166 (50.8)	
Heterosexual	466 (6.1)	25 (6.3)	32 (9.8)	
IDU	2235 (29.2)	118 (29.6)	116 (35.5)	
Unknown	266 (3.5)	20 (5.0)	13 (4.0)	
**Spanish Origin, n (%)**	4739 (61.8)	230 (54.2)	168 (51.4)	<0.001
**Education level, n (%)**				<0.001
No studies or compulsory	2217 (28.9)	148 (34.9)	111 (33.9)	
Upper secondary or university	4097 (53.5)	226 (53.3)	182 (55.7)	
Unknown	1351 (17.6)	50 (11.8)	34 (10.4)	
**AIDS diagnosis, n (%)**	873 (11.4)	48 (12.0)	46 (14.1)	0.31
**HCV positive ever, n (%)**	680 (8.9)	41 (10.3)	44 (13.5)	0.001
**Maximum HIV-1 RNA, n (%)**				0.04
**<100000 copies/mL**	4749 (62.0)	262 (61.8)	200 (61.2)	
**≥100000 copies/mL**	2580 (33.7)	153 (36.1)	120 (36.7)	
**Unknown**	336 (4.4)	9 (2.1)	7 (2.1)	
**Time from ART initiation to virologic suppression (years), median (IQR)**	0.3 (0.2 – 0.5)	0.3 (0.2 – 0.5)	0.3 (0.2 – 0.5)	0.03

**Time from virologic suppression (years) to ART switch, median (IQR)**	-	3.4 (1.8 – 5.9)	2.7 (1.3 – 4.2)	0.012
**Nadir CD4 cell count (cells/μL), median (IQR)**	321 (198 – 463)	290 (159 – 417)	270 (151 – 366)	<0.001
**Time from ART initiation to censoring, median [years (IQR)]**	3.8 (2.0 – 6.5)	6.0 (3.8 – 8.4)	7.3 (5.3-9.5)	<0.001
**Number of HIV RNA determinations from ART initiation to last study contact median (IQR)]**	13 (7 – 20)	20 (14 – 27)	23 (16 – 30)	<0.001

Abbreviations: 3DR, triple therapy; 2DR, 2-drug combinations; MT, monotherapy; ART, antiretroviral therapy; IDU, injecting drug use; MSM, men who have sex with men.

Table S2. Rate of severe non-AIDS events during the follow-up

	3DR	2DR	MT
	N=90	N=58	N=23
**Mortality**	0 (0)	0 (0)	0 (0)
**Cardiometabolic (N, %)**			
Diabetes	2 (2)	1 (2)	0 (0)
Myocardial infarction	1 (1)	0 (0)	1 (4)
Stroke	0 (0)	2 (3)	0 (0)
**Non-AIDS malignancies (N, %)**	0 (0)	2 (3)	0 (0)
**Visceral leishmaniasis (N, %)**	1 (1)	0 (0)	0 (0)

Table S3. Adjusted coefficients of inflammatory biomarker changes by ART strategy (MT vs. 3DR) at year 0-3 and 3-8 from virologic suppression.

	Coefficient	95% CI	p
**Years 0-3 of viral suppression**			
IL-6 (log10)	0.066	-0.036, 0.167	0.204
hs-CRP	0.067	-0.034, 0.170	0.194
D-dimers	-27.256	-45.877, -8.636	**0.004**
IFABP	-0.973	-2-23, 0.292	0.132
sCD163 (log10)	0.067	0.004, 0.131	0.038
sCD14	0.102	-0.122, 0.326	0.373
**Years 3-8 of viral suppression**			
IL-6 (log10)	-0.027	-0.159, 0.106	0.690
hs-CRP	0.052	0.051, 0.155	0.328
D-dimers	26.179	9.806	42.552
IFABP	0.453	0.598, 1.506	0.398
sCD163 (log10)	0.041	-0.022, 0.103	0.201
sCD14	0.114	0-069, 0.297	0.225

*Reference category: MT

Abbreviations: ART, antiretroviral therapy; 3DR, triple therapy; MT, monotherapy; CI, confidence interval; INSTI, integrase strand transfer inhibitor; NRTI, nucleoside reverse transcriptase inhibitor; NNRTI, nonnucleoside reverse transcriptase inhibitor; PI, protease inhibitor

Supplemental figures

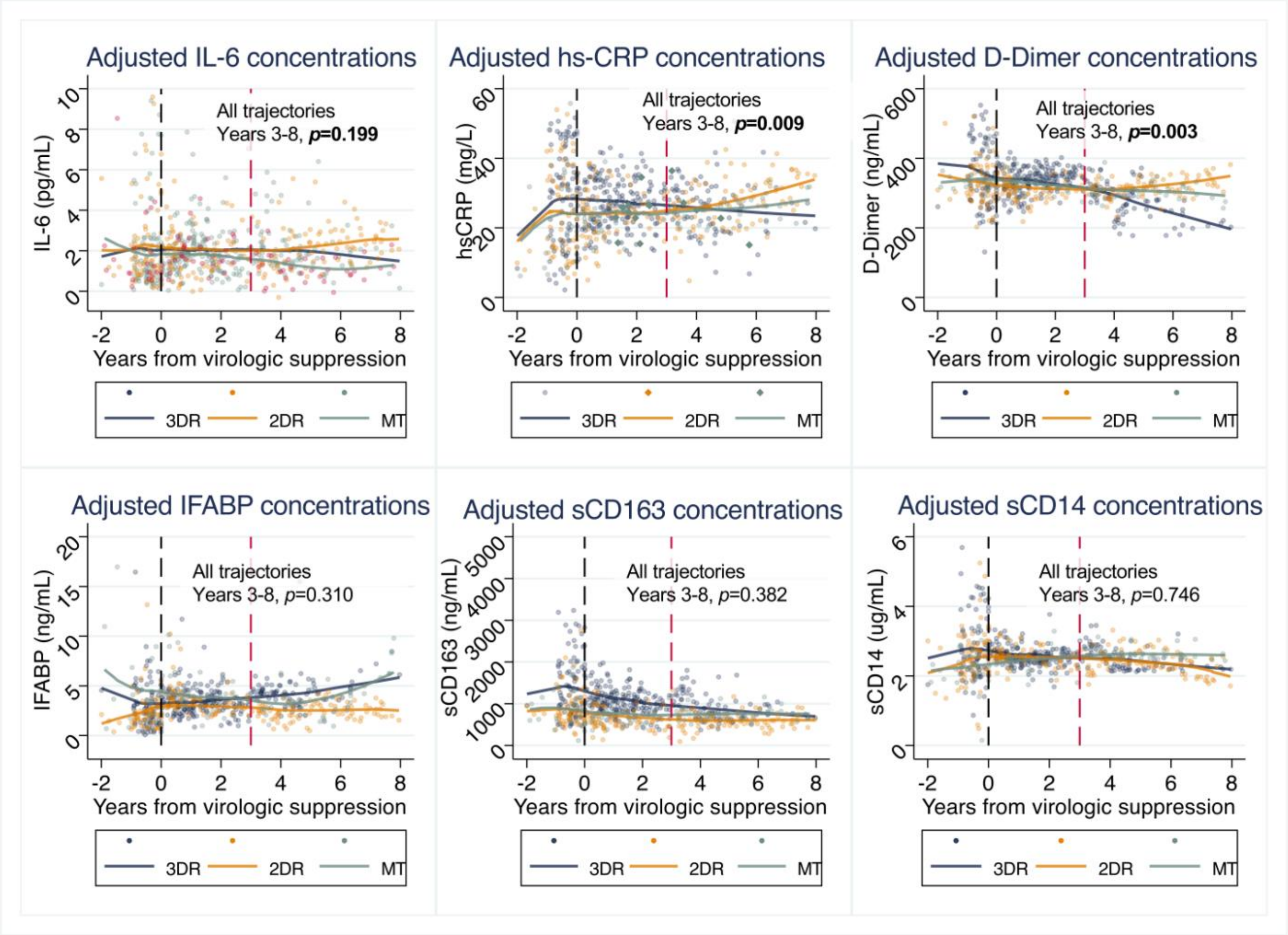

Figure S1. Effects of switch to MT on inflammatory biomarkers compared to remaining on 3DR. Panels represent the values predicted in piecewise linear mixed models at intervals between years 0-1, 1-2, 3-4 and 4-8, adjusted for age, sex, country of origin, mode of transmission, educational level, maximum HIV RNA, previous AIDS, nadir CD4, and pre-ART biomarker value. Lines represent predicted mean values and dots the individual observations. The P values computed using Wald tests represent the global-group comparison of biomarker trajectories from year 3 to 8. The tables piecewise comparisons for the periods 0-3 and 3-8, are provided in **Table S2**.
